# Time required to achieve optimum viral load suppression with Ravidasvir/sofosbuvir in chronic hepatitis C patients with or without compensated cirrhosis

**DOI:** 10.1038/s41598-025-99665-7

**Published:** 2025-04-25

**Authors:** Nor Asiah Muhamad, Izzah Athirah Rosli, Nur Hasnah Maamor, Rozainanee Mohd Zain, Fatin Norhasny Leman, Huan-Keat Chan, Muhammad Radzi Abu Hassan, Shahnaz Murad

**Affiliations:** 1 Sector for Evidence-Based Healthcare, National Institutes of Health, Ministry of Health, Selangor, Malaysia; 2 Institute for Medical Research, National Institutes of Health, Ministry of Health, Selangor, Malaysia; 3 Office of the Director General of Health, Ministry of Health, Putrajaya, Malaysia; 4 Former Office of the Deputy Director General of Health (Research and Technical Support), Ministry of Health, Putrajaya, Malaysia

**Keywords:** Ravidasvir, Sofosbuvir, Hepatitis, Time-to-event, Virologic response, STORM-C-1, Hepatitis C, Combination drug therapy

## Abstract

A study indicated that ravidasvir (RDV) has excellent safety and tolerability when used with sofosbuvir (SOF) to treat chronic HCV infection. The aim of this study was to determine the time taken by RDV/SOF to achieve optimum viral load suppression in chronic hepatitis C patients with or without compensated cirrhosis. Data from the open-label, multicentre, single-arm, phase II/III clinical trial (STORM-C-1) were utilized. Time‒to-event analysis via Kaplan–Meier curves was performed to determine the time required to achieve optimum viral load suppression in both the cirrhotic and noncirrhotic groups. Multivariate logistic regression analyses were performed to identify potential predictors of achieving suppression within four and eight weeks. The time to achieve optimum viral load suppression ranged from six to 85 days and from five to 148 days among noncirrhotic and cirrhotic patients, respectively. Among noncirrhotic patients, 80.6% achieved optimum viral load suppression within 4 weeks, and 92.6% achieved this within 8 weeks. Among cirrhotic patients, 76.1% and 90.4% achieved optimum viral load suppression within 4 and 8 weeks, respectively. Notably, optimum viral load suppression differs from sustained virological response (SVR12), which is defined as undetectable HCV RNA 12 weeks after treatment completion. While the study demonstrates promising early viral suppression, it does not evaluate the efficacy of a shortened regimen. Further research is needed to assess whether shorter treatment durations maintain high SVR12 rates without compromising treatment success.

## Introduction

Hepatitis C virus (HCV) infection is a global public health threat that currently affects more than 50 million people^1^. Single-stranded RNA viruses were discovered in 1989^2^. The virus is transmitted mainly through blood, and most infections result from exposure to contaminated blood, which can occur due to inadequate health care, unscreened blood transfusions, injection drug use, sexual activities that involve blood exposure, and unsafe injection practices, especially when the needle or other medical equipment is not sterile^1,3^. HCV infection can cause cirrhosis and even hepatocellular carcinoma over the long term, eventually leading to mortality^4–7^. The World Health Organization (WHO) estimated that approximately one million new cases occur every year^1^. According to the Centers for Disease Control and Prevention (CDC), the African region, particularly Egypt, has the highest prevalence of chronic HCV infection^8^. Compared to high-income countries, low-middle-income countries (LMICs) generally have a higher prevalence of HCV infection, often exceeding 5.0%^9^.

Eight HCV genotypes and 86 subtypes have been identified, with genotypes 1 (44%), 3 (25%), and 4 (15%) being the most common^10^ Blanch et al. reported that genotype 1 is more common in high-income countries, whereas genotype 3 predominates in LMICs^11^. Genotype 3 infection is associated with a poorer response to direct-acting antivirals (DAAs), primarily in patients with cirrhosis, prior peginterferon treatment, and hepatic steatosis, increasing the risk of progression to cirrhosis and hepatocellular carcinoma^12,13^. Additionally, the IL-28B genotype influences the treatment response. Compared with HCV-infected individuals, those with the CC genotype exhibit a stronger immune response than do those with non-CC (CT or TT) genotypes^14^.

Conventional antiviral therapies for HCV, mainly interferon (INF) and ribavirin (RBV), have reported limited benefits and significant adverse events^15,16^. Despite being the standard therapy^17^, interferon-based treatment has been replaced by DAAs in recent years. DAAs have revolutionized the treatment of chronic hepatitis C virus (HCV) infection by directly targeting viral replication mechanisms. Sofosbuvir (SOF), an NS5B polymerase inhibitor, has demonstrated efficacy in multiple studies when combined with other DAAs^18,19^. Recently, the combination of ravidasvir (RDV), a potent NS5A inhibitor and sofosbuvir has shown its excellent efficacy and safety^17^. Their combination synergistically blocks HCV replication, prevents viral assembly, and enhances viral clearance. Compared to earlier DAAs, the RDV/SOF regimen offers several advantages. Unlike some DAAs that are genotype-specific, RDV/SOF is pangenotypic, as it is effective across all HCV genotypes, making it particularly useful in regions with diverse genotype distributions^20^. While some NS5A inhibitors like daclatasvir are associated with resistance mutations, particularly in genotype 3^21^, RDV has demonstrated a higher genetic barrier to resistance, reducing the risk of treatment failure^17^. The emergence of more pangenotypic DAAs represents a significant advancement in HCV treatment, offering enhanced safety profiles and reduced mortality rates^22^.

The recent STORM-C-1 trial^13^ evaluated the efficacy and safety of treatment with RDV/SOF, which demonstrated efficacy and good tolerability in patients with chronic HCV infection, particularly those without cirrhosis or with compensated cirrhosis. These findings add to the literature by suggesting RDV/SOF as an efficacious and safe antiviral option in these patients. The interim analysis indicated that over 90% of the patients achieved viral load suppression (HCV RNA < 15 IU/mL) much earlier than 12–24 weeks, with 97% achieving a sustained virological response 12 weeks after treatment (SVR12). In fact, some studies have advocated for a shorter duration of DAA therapy, providing evidence of its efficacy, financial benefits and patient compliance^23–25^. Therefore, a time-to-event study nested in the STORM-C-1 trial was conducted to determine the time to achieve optimum viral load suppression with RDV/SOF in patients with chronic hepatitis C infection, particularly those without cirrhosis or with compensated cirrhosis, and to determine the proportion of patients who achieved suppression at the earliest, within four and eight weeks of treatment, as well as their potential predictors.

## Materials and methods

### Study design

We utilized data from an open-label, multicentre, international, single-arm, phase II/III clinical trial (STORM-C-1), which was conducted from 2016 to 2020 and is detailed elsewhere^13^. The objective of the original STORM-C-1 study was to evaluate the efficacy, adverse events, and pharmacokinetics of 12- and 24-week treatments with RDV/SOF in people with HCV infection in Malaysia and Thailand. Patients without cirrhosis received 200 mg of RDV and 400 mg of SOF once daily for 12 weeks, whereas patients with cirrhosis received the same regimen for 24 weeks. The trial was registered with ClinicalTrials.gov (NCT02961426, dated 11/11/2016) and the National Medical Research Register of Malaysia (NMRR-16-747-29183). The trial was conducted in accordance with Good Clinical Practice guidelines, the Declaration of Helsinki, and applicable local regulations. The trial protocol was reviewed and approved by the Medical Research and Ethics Committee (MREC) of the Ministry of Health (MOH) Malaysia and the Ethical Review Committee for Research in Human Subjects, Ministry of Public Health (ECMOPH) Thailand. All patients’ written informed consents were obtained.

### Study population

We included all patients from the STORM-C-1 study who were both treatment-naïve and treatment-experienced individuals, aged 18–69 years, of both sexes, had a body mass index (BMI) between 18 and 35 kg/m², had chronic HCV infection of any genotype, and were without cirrhosis or with compensated cirrhosis. Patients with decompensated cirrhosis, hepatocellular carcinoma, hepatitis B virus coinfection, end-stage renal disease, or a history of treatment with any NS5A inhibitor were excluded from the original study^13^. Of the 603 patients included in the STORM-C-1 study, only 580 were eligible for this study. The remaining 23 patients were excluded for reasons including incomplete treatment and loss to follow-up.

### Outcomes

The primary outcome for this study was the time to achieve optimum viral load suppression, defined as achieving HCV RNA < 15 IU/mL. The secondary outcomes were the proportion of patients who achieved suppression at the earliest, within four and eight weeks of treatment, also known as early viral suppression in this study, as well as the potential predictors associated with the time to achieve the suppression.

### Data analysis

We conducted time‒to-event analysis via Stata/IC statistical software version 16 (StataCorp LLC, College Station, TX, USA). An event was defined as achieving optimum viral load suppression (HCV RNA < 15 IU/mL). A Kaplan–Meier curve was constructed to determine the time to viral load suppression for treatment with RDV/SOF. The log-rank test was used to compare the time to achieve optimum viral load suppression across different variables, including sex, age groups, HCV genotypes, IL-28B genotypes, the presence of HIV coinfection, previous interferon treatment exposure, injection drug use, the use of concomitant medication, and the presence of comorbidities. Multiple Cox proportional regression analysis was conducted to estimate the hazard ratios (HRs) for each variable, indicating the relative risk of achieving optimum viral load suppression. The proportional hazards assumption in the Cox regression model was examined using log-log survival plots and time-dependent covariates. To ensure more reliable estimation, both Cox models for noncirrhosis and cirrhosis groups were stratified by sex and age due to their strong violation to the assumption. Additionally, other covariates that violate the assumption were modelled as time-dependent covariates to account for their non-proportional effects. A descriptive analysis was conducted to determine the proportion of patients who achieved early viral suppression, within four and eight weeks of treatment. Multivariate logistic regression analyses were performed to identify factors associated with early viral suppression at four and eight weeks. A *p* value of < 0.05 was considered to indicate statistical significance.

## Results

### Patient characteristics

Among the 580 patients with HCV infection included in this study, 350 (60.3%) did not have cirrhosis, and 230 (39.7%) had compensated cirrhosis. Table 1 shows the patient characteristics. Most of them were male (78.1%), with a mean age of 46.8 years (SD: 10.5 years; range: 20–67 years). Genotype 1 and 3 infections predominated in both noncirrhotic and cirrhotic patients. A high viral load (> 800,000 IU/mL) was observed in 396 patients (68.3%) at baseline. CC (77.1%) was the most common IL-28B genotype. Over 30% of patients had HIV coinfection, whereas 79.8% were interferon treatment-naïve. Injection drug use was reported in 46.6% of these patients. Compared with noncirrhotic patients, cirrhotic patients had greater mean baseline liver stiffness. Over 90% of the patients reported the use of concomitant medications, whereas 17.1% had comorbidities.

**Table 1:** Baseline characteristics of HCV patients receiving RDV/SOF. (*N* = 580).

	Overall (*N* = 580)	Noncirrhosis (*n* = 350)	Cirrhosis (*n* = 230)
Sex			
Female	127 (21.9)	77 (22.0)	50 (21.7)
Male	453 (78.1)	273 (78.0)	180 (78.3)
Age group (years)	46.8 ± 10.5	44.3 ± 10.5	50.8 ± 9.3
< 45	239 (41.2)	179 (51.1)	60 (26.1)
≥ 45	341 (58.8)	171 (48.9)	170 (73.9)
Country of treatment			
Malaysia	381 (65.7)	178 (50.9)	203 (88.3)
Thailand	199 (34.3)	172 (49.1)	27 (11.7)
HCV genotype			
1	230 (39.7)	162 (46.3)	68 (29.6)
2	3 (0.5)	3 (0.9)	0
3	290 (50.0)	135 (38.6)	155 (67.4)
6	57 (9.8)	50 (14.3)	7 (3.0)
Baseline HCV RNA (IU/mL)			
< 800,000	184 (31.7)	104 (29.7)	80 (34.8)
≥ 800,000	396 (68.3)	246 (70.3)	150 (65.2)
IL-28B genotype			
CC	447 (77.1)	266 (76.0)	181 (78.7)
CT + TT	133 (22.9)	84 (24.0)	49 (21.3)
HIV coinfection			
No	396 (68.3)	208 (59.4)	188 (81.7)
Yes	184 (31.7)	142 (40.6)	42 (18.3)
Previous interferon treatment exposure			
Treatment-experienced	117 (20.2)	70 (20.0)	47 (20.4)
Treatment-naïve	463 (79.8)	280 (80.0)	183 (79.6)
Injection drug use			
No	310 (53.4)	187 (53.4)	123 (53.5)
Yes	270 (46.6)	163 (46.6)	107 (46.5)
Liver stiffness (kPa)	13.7 ± 10.6	7.3 ± 2.4	23.5 ± 10.8
Use of concomitant medications			
No	54 (9.3)	41 (11.7)	13 (5.7)
Yes	526 (90.7)	309 (88.3)	217 (94.3)
Comorbidities			
No	481 (82.9)	302 (86.3)	179 (77.8)
Yes	99 (17.1)	48 (13.7)	51 (22.2)
Common comorbidities			
Diabetes mellitus	48 (8.3)	19 (5.4)	29 (12.6)
Obesity	29 (5.0)	9 (2.6)	20 (8.7)
NAFLD	30 (5.2)	17 (4.9)	13 (5.7)

Data are presented as frequencies (percentages) or means ± standard deviations.

### Time to achieve the optimum viral load suppression

**Fig. 1:**
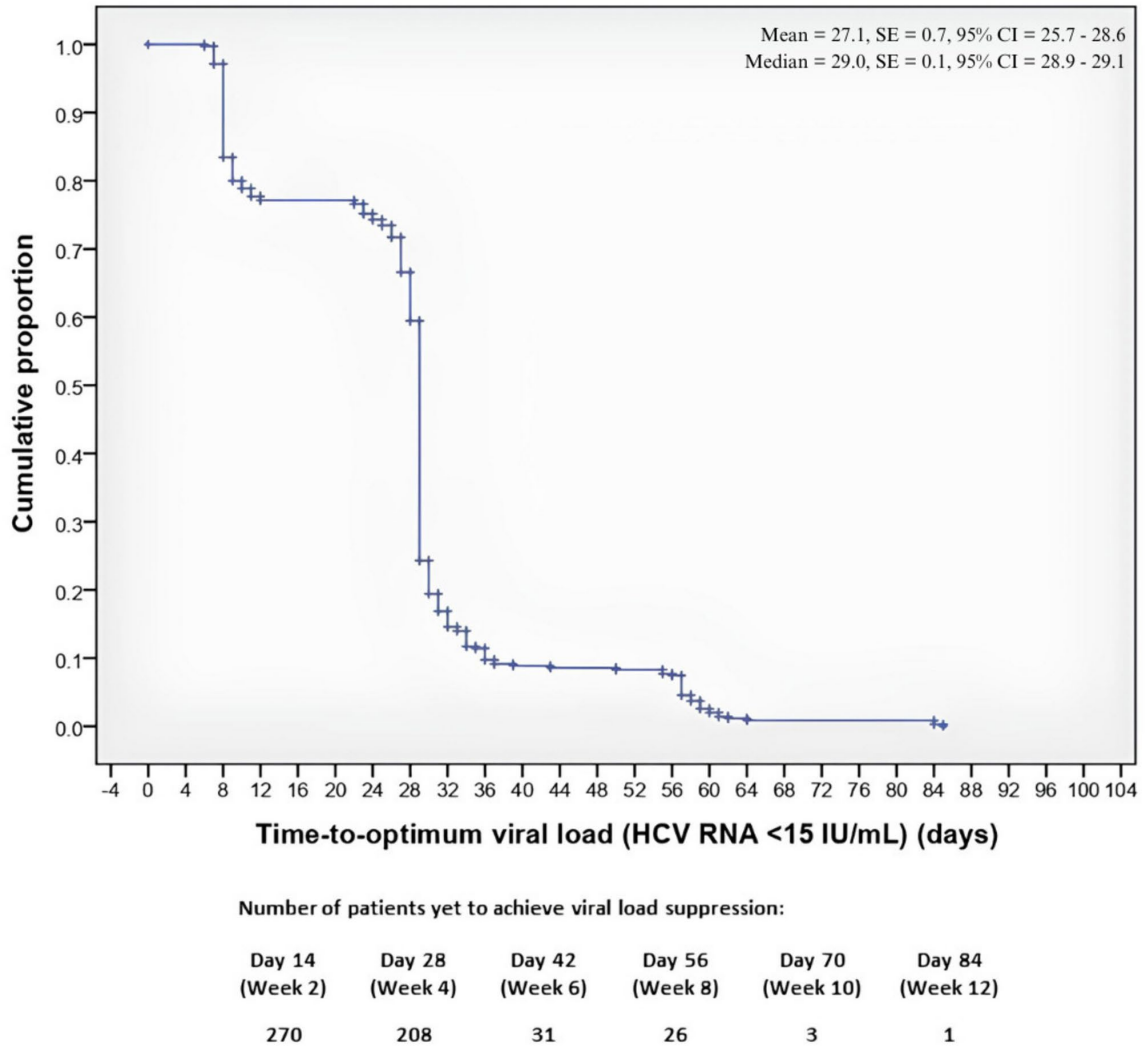
Kaplan-Meier curve showing the time to achieve optimum viral load suppression (< 15 IU/mL) among noncirrhotic patients treated with RDV/SOF.

In the analysis of 350 noncirrhotic patients, the shortest and longest times taken to achieve optimum viral load suppression (HCV RNA < 15 IU/mL) were six and 85 days, respectively. The time taken for 50% of noncirrhotic patients to achieve suppression was 29 days. The Fig. 1 shows the time to achieve the optimum viral load in noncirrhotic patients. The log-rank analysis revealed no significant differences in the time to achieve optimum viral load suppression across sex (*p* = 0.223), age groups (*p* = 0.358), HCV genotypes (*p* = 0.083), IL-28B genotypes (*p* = 0.342), the presence of HIV coinfection (*p* = 0.565), previous interferon treatment exposure (*p* = 0.158), injection drug use (*p* = 0.518), the use of concomitant medications (*p* = 0.121), or the presence of comorbidities (*p* = 0.666).

Table 2 presents the results of multiple Cox proportional hazards regression analysis, evaluating factors associated with achieving optimal viral load suppression among noncirrhotic patients. HCV genotype 2 was significantly associated with a higher likelihood of achieving viral suppression (adjusted HR = 1.854, 95% CI: 1.142–3.702, *p* = 0.020). Patients with the IL-28B CC genotype had a significantly lower likelihood of achieving viral suppression (adjusted HR = 0.002, 95% CI: 0.000–0.007, *p* = 0.001), suggesting a strong negative impact of this genotype on treatment response. Injection drug use was also significantly associated with a higher likelihood of achieving viral suppression (adjusted HR = 1.232, 95% CI: 1.027–1.487, *p* = 0.023). Patients who were treatment-naïve had a much higher likelihood of achieving viral suppression (adjusted HR = 4.7E + 4, 95% CI: 3.8E + 3–3.5E + 7, *p* = 0.001). However, this extreme HR suggests a possible confounding effect, likely influenced by IL-28B genotype distribution among treatment-naïve and experienced patients. For the time-dependent covariates, the effect of both IL-28B genotype and previous interferon exposure on viral suppression significantly decreased over time (*p* < 0.05), indicating that their influence is stronger in the earlier phases of treatment but diminishes as treatment progresses.

**Table 2:** Multiple Cox proportional hazards regression analysis of factors associated with achieving optimum viral load suppression among noncirrhotic patients, stratified by sex and age group.

	N of events^a^ (*N* = 230)	Adjusted HR	95% CI	*p* value^b^
HCV genotype				
6 (ref)	50 (14.3)	1.000	–	–
1	162 (46.3)	1.151	0.963–1.432	0.153
2	3 (0.9)	1.854	1.142–3.702	**0.020**
3	135 (38.6)	0.986	0.797–1.241	0.902
IL-28B genotype				
CT + TT (ref)	84 (24.0)	1.000	–	–
CC	266 (76.0)	0.002	0.000–0.007	**0.001**
HIV coinfection				
No (ref)	208 (59.4)	1.000	–	–
Yes	142 (40.6)	1.128	0.935–1.359	0.212
Previous interferon treatment exposure				
Treatment-experienced (ref)	70 (20.0)	1.000	–	–
Treatment-naïve	280 (80.0)	4.7E + 4	3.8E + 3–3.5E + 7	**0.001**
Injection drug use				
No (ref)	187 (53.4)	1.000	–	–
Yes	163 (46.6)	1.232	1.027–1.487	**0.023**
Use of concomitant medications				
No (ref)	41 (11.7)	1.000	–	–
Yes	309 (88.3)	0.918	0.659–1.358	0.630
Comorbidities				
No (ref)	302 (86.3)	1.000	–	–
Yes	48 (13.7)	0.961	0.703–1.247	0.768
IL-28B genotypes * Log Time	–	0.144	0.068–0.221	**0.001**
Previous interferon treatment exposure * Log time	–	0.044	0.007–0.091	**0.001**

^a^Number of patients achieved optimum viral load (HCV RNA < 15 IU/mL) within 24 weeks of treatment. Data are presented as frequencies (percentages).

^b^Significant at *p* < 0.05 (bold).

**Fig. 2:**
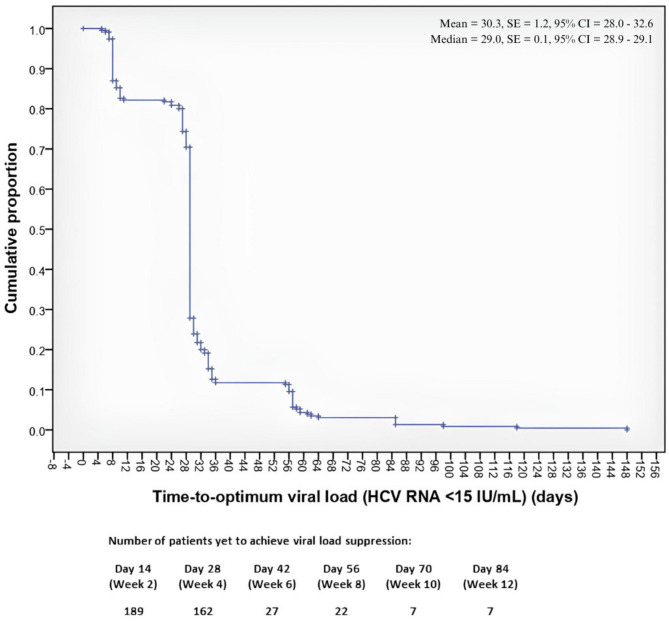
Kaplan-Meier curve showing the time to achieve optimum viral load suppression (< 15 IU/mL) among cirrhotic patients treated with RDV/SOF.

**Fig. 3:**
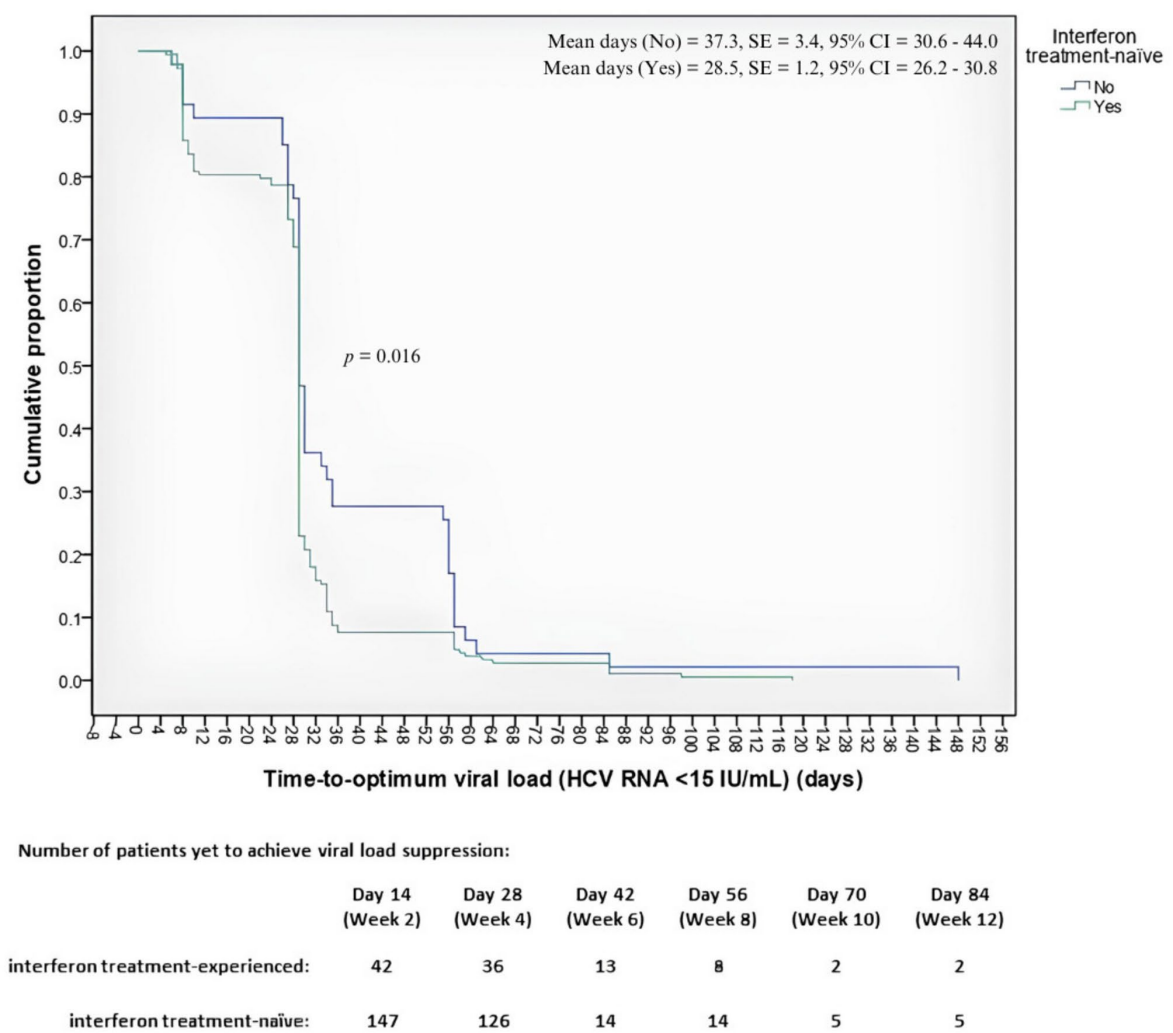
Log-rank analysis showing the time to achieve optimum viral load between interferon treatment-naïve and interferon treatment-experienced patients with cirrhosis treated with RDV/SOF.

For 230 cirrhotic patients, the shortest time taken to achieve optimum viral load suppression was five days, and the longest time was 148 days. The time taken for 50% of the patients with cirrhosis to achieve optimum viral load suppression was 29 days. The time to optimum viral load suppression in cirrhotic patients is presented in Fig. 2. The log-rank analysis revealed no significant differences across sex (*p* = 0.947), age groups (*p* = 0.067), HCV genotypes (*p* = 0.231), IL-28B genotypes (*p* = 0.147), the presence of HIV coinfection (*p* = 0.114), injection drug use (*p* = 0.326), the use of concomitant medication (*p* = 0.653), or the presence of comorbidities (*p* = 0.449). However, there was a significant difference in the time to achieve optimum viral load suppression between interferon-naïve patients and interferon-experienced patients (*p* = 0.016) **(**Fig. 3**)**.

Table 3 presents the results of multiple Cox proportional hazards regression analysis, evaluating factors associated with achieving optimal viral load suppression among cirrhotic patients. Patients with the IL-28B CC genotype had a significantly lower likelihood of achieving viral suppression (adjusted HR = 0.001, 95% CI: 0.000–0.001, *p* = 0.002), indicating a strong negative impact of this genotype on treatment response. For the time-dependent covariates, the effect of IL-28B genotype on viral suppression significantly decreased over time (*p* = 0.002), suggesting that its initial impact on viral suppression decreases over the course of treatment. Previous interferon treatment exposure and use of concomitant medication were excluded from the Cox model; the former due to severe multicollinearity and the latter due to extreme group imbalance.

**Table 3:** Multiple Cox proportional hazards regression analysis of factors associated with achieving optimum viral load suppression among cirrhotic patients, stratified by sex and age group.

	*N* of events^a^ (*N* = 230)	Adjusted HR	95% CI	*p* value^b^
HCV genotype				
6 (ref)	7 (3.0)	1.000	–	–
1	68 (29.6)	1.352	0.721–2.208	0.245
3	155 (67.4)	1.122	0.595–1.777	0.613
IL-28B genotype				
CT + TT (ref)	49 (21.3)	1.000	–	–
CC	181 (78.7)	0.001	0.000–0.001	**0.002**
HIV coinfection				
No (ref)	188 (81.7)	1.000	–	–
Yes	42 (18.3)	1.106	0.304–1.595	0.508
Injection drug use				
No (ref)	123 (53.5)	1.000	–	–
Yes	107 (46.5)	0.920	0.742–1.121	0.382
Comorbidities				
No (ref)	179 (77.8)	1.000	–	–
Yes	51 (22.2)	1.000	0.795–1.226	0.998
IL-28B genotypes * Log Time	–	0.081	0.047–0.103	**0.002**

^a^Number of patients achieved optimum viral load (HCV RNA < 15 IU/mL) within 24 weeks of treatment. Data are presented as frequencies (percentages).

^b^Significant at *p* < 0.05 (bold)

### Early viral suppression within 4 and 8 weeks

The proportions of noncirrhotic patients who achieved optimum viral load suppression within four and eight weeks of treatment were 40.6% (142/350) and 92.6% (324/350), respectively. Moreover, the percentages of patients with cirrhosis who achieved suppression within four and eight weeks were 29.6% (68/230) and 90.4% (208/230), respectively. A multivariate logistic regression analysis was performed to identify potential predictors of early viral load suppression within four and eight weeks among noncirrhotic patients **(**Table 4**)**. Compared with treatment-experienced patients, treatment-naïve patients are significantly more likely to achieve optimum viral load suppression within four weeks (OR 1.909, 95% CI 1.032–3.529; *p* = 0.039). Compared with nondrug users, injection drug users are also found to be significantly more likely to achieve suppression within four weeks (OR 1.721, 95% CI 1.024–2.893, *p* = 0.040). A multivariate logistic regression was also performed among cirrhotic patients to identify potential predictors to achieve optimum viral load suppression within four and eight weeks **(**Table 5**)**. No significant associations were found between any of the variables (all *p* > 0.05), except that cirrhotic patients with HIV coinfection were significantly more likely to achieve optimum viral load suppression within four weeks than patients without HIV coinfection (OR 2.405, 95% CI 1.135–5.097, *p* = 0.022), and treatment-naïve cirrhotic patients are significantly more likely to achieve early viral load suppression within eight weeks as compared to treatment-experienced patients (OR 2.970, 95% CI 1.052–8.386, *p* = 0.040).

**Table 4:** Multivariate logistic regression analysis of the likelihood of achieving optimum viral load suppression within four and eight weeks among noncirrhotic patients. (*N* = 350).

	Achieve optimum viral load suppression within four weeks			Achieve optimum viral load suppression within eight weeks		
	OR	95% CI	*p* value^a^	OR	95% CI	*p* value^a^
Sex						
Female (ref)	1.000	–	–	1.000	–	–
Male	0.563	0.300–1.058	0.074	0.906	0.278–2.955	0.870
Age group (years)						
< 45 (ref)	1.000	–	–	1.000	–	–
≥ 45	0.812	0.517–1.273	0.364	2.152	0.906–5.109	0.082
HCV genotype						
6 (ref)	1.000	–	–	1.000	–	–
1	1.067	0.540–2.107	0.852	1.420	0.452–4.467	0.548
2	4.792	0.371–61.97	0.230	19E07	0	0.999
3	1.290	0.636–2.613	0.480	2.637	0.734–9.478	0.137
IL-28B genotype						
CT + TT (ref)	1.000	–	–	1.000	–	–
CC	0.933	0.543–1.603	0.802	1.439	0.557–3.721	0.453
HIV coinfection						
No (ref)	1.000	–	–	1.000	–	–
Yes	1.357	0.819–2.249	0.236	1.636	0.644–4.160	0.301
Previous interferon treatment exposure						
Treatment-experienced (ref)	1.000	–	–	1.000	–	–
Treatment-naïve	1.909	1.032–3.529	**0.039**	0.902	0.296–2.748	0.856
Injection drug use						
No	1.000	–	–	1.000	–	–
Yes	1.721	1.024–2.893	**0.040**	1.009	0.400–2.546	0.985
Use of concomitant medications						
No	1.000	–	–	1.000	–	–
Yes	0.657	0.314–1.374	0.264	0.436	0.088–2.153	0.308
Comorbidities						
No	1.000	–	–	1.000	–	–
Yes	0.819	0.421–1.596	0.558	0.802	0.248–2.597	0.713

^a^Multivariate logistic regression, ‘Enter’ method, significant at *p* < 0.05 (bold).

**Table 5:** Multivariate logistic regression analysis of the likelihood of achieving optimum viral load suppression within four and eight weeks among cirrhotic patients. (*N* = 230).

	Achieve optimum viral load suppression within four weeks			Achieve optimum viral load suppression within eight weeks		
	OR	95% CI	*p* value^a^	OR	95% CI	*p* value^a^
Sex						
Female (ref)	1.000	–	–	1.000	–	–
Male	0.800	0.353–1.812	0.593	0.992	0.265–3.711	0.991
Age group (years)						
< 45 (ref)	1.000	–	–	1.000	–	–
≥ 45	0.816	0.412–1.619	0.561	0.830	0.246–2.799	0.764
HCV Genotype						
6 (ref)	1.000	–	–	1.000	–	–
1	3.800	0.417–34.63	0.236	7.119	0.493–102.9	0.150
2	-	–	–	–	–	–
3	3.070	0.349–27.00	0.312	1.539	0.156–15.15	0.712
IL-28B genotype						
CT + TT (ref)	1.000	–	–	1.000	–	–
CC	0.872	0.423–1.798	0.711	0.312	0.065–1.496	0.145
HIV coinfection						
No (ref)	1.000	–	–	1.000	–	–
Yes	2.405	1.135–5.097	**0.022**	2.284	0.483–10.81	0.297
Previous interferon treatment exposure						
Treatment-experienced (ref)	1.000	–	–	1.000	–	–
Treatment-naïve	1.593	0.716–3.541	0.254	2.970	1.052–8.386	**0.040**
Injection drug use						
No	1.000	–	–	1.000	–	–
Yes	0.677	0.339–1.355	0.271	0.755	0.250–2.277	0.617
Use of concomitant medications						
No	1.000	–	–	1.000	–	–
Yes	1.174	0.323–4.264	0.807	1.919	0.192–19.22	0.579
Comorbidities						
No	1.000	–	–	1.000	–	–
Yes	0.485	0.222–1.062	0.070	0.577	0.195–1.707	0.320

^a^Multivariate logistic regression, ‘Enter’ method, significant at *p* < 0.05 (bold).

### Relationship between early viral suppression and SVR12

The relationship between early viral suppression and SVR12 was analyzed. While optimum viral load suppression refers to achieving HCV RNA < 15 IU/mL during treatment, SVR12 is defined as undetectable HCV RNA 12 weeks after treatment completion, serving as the standard measure of treatment success. Among noncirrhotic patients who achieved viral suppression within 4 weeks, 96.5% (137/142) subsequently achieved SVR12, compared to 97.6% (203/208) of those who took longer than 4 weeks to achieve suppression (*p* = 0.538). Among those who achieved viral suppression within 8 weeks, 96.9% (314/324) attained SVR12, while all patients (26/26) who took longer than 8 weeks also achieved SVR12 (*p* = 0.363). Among cirrhotic patients, all individuals who achieved viral load suppression within 4 weeks (68/68) later achieved SVR12, compared to 98.1% (159/162) of those who took longer than 4 weeks (*p* = 0.259). For those who achieved suppression within 8 weeks, 99.0% (206/208) attained SVR12, compared to 95.5% (21/22) of those who took longer than 8 weeks (*p* = 0.159).

## Discussion

The STORM-C-1 study^13^ demonstrated the efficacy of 12- and 24-week treatments with RDV/SOF, establishing this combination as a recommended treatment regimen for chronic HCV infection. The present analysis adds to the literature, presenting the time to achieve optimum viral load suppression (HCV RNA concentration < 15 IU/mL) among HCV-infected patients treated with RDV/SOF. The shortest duration recorded to suppress the HCV load below the optimum concentration was five and six days for patients with and without cirrhosis, respectively. Additionally, the majority of the patients without cirrhosis (80.6%) and with compensated cirrhosis (76.1%) achieved optimum viral load suppression within four weeks, and more than 90% of the patients in both groups achieved optimum viral load suppression within eight weeks of treatment. Although the primary objective of this study was to evaluate the time to achieve optimum viral load suppression, adverse events (AEs) were also observed. The most commonly reported AEs were pyrexia, cough, headache, upper respiratory tract infection (URTI), and lethargy. Only 5.2% of patients experienced grade 3 or higher toxicity, all of whom fully recovered. These findings indicate that viral suppression often occurs early in treatment, raising the possibility that a shorter regimen could be effective. However, this study did not assess the efficacy of a shortened RDV/SOF regimen, hence further randomized controlled trials are necessary to determine whether reducing treatment duration maintains high cure rates without compromising the treatment success.

In this study, we defined optimum viral load suppression as HCV RNA < 15 IU/mL, which aligns with the lower limit of detection (LLOD) of many contemporary HCV RNA assays. While various thresholds have been used in clinical practice and research, the clinical relevance of these small differences remains debatable. The European Association for the Study of the Liver (EASL)^26^ and the American Association for the Study of Liver Diseases (AASLD) guidelines consider HCV RNA levels below LLOD as indicating viral clearance, though the specific threshold may vary depending on the sensitivity of the assay used. Recent clinical trials evaluating direct-acting antivirals have commonly used thresholds of < 12–15 IU/mL, making our results comparable with contemporary literature^27–29^. The selected threshold of < 15 IU/mL represents a clinically meaningful marker of viral suppression that balances assay sensitivity with practical clinical utility.

The present study identified few key predictors of early viral suppression, including treatment-naïve, injection drug use, and co-infected with HIV. Treatment-naïve patients were found to be more likely to achieve viral suppression within four and eight weeks in noncirrhotic and cirrhotic patients. Nagaty and Ekram^30^ observed that treatment-naïve patients treated with sofosbuvir plus ribavirin achieved a high response rate, with 100% of them achieving early virological response at 4 weeks, whereas some treatment-experienced patients had a slower response. In contrast, Sarrazin^31^ found that some treatment-experienced patients achieved viral suppression at a similar rate to treatment-naïve patients when using optimized DAA regimens. These findings were possibly due to the absence of prior drug resistance or treatment exposure, which aligns with the understanding that the NS5B RNA polymerase and NS5A protein are highly effective antiviral targets in untreated individuals^32^.

Besides treatment-naïve, injection drug use also was identified as significant factor associated with early viral suppression in this study. Eckhardt^33^ stated that injection drug users treated with DAAs had high rates of early viral suppression and sustained virological response, comparable or better than non-drug users. In contradict, Vallet-Pichard and Pol^34^ found that injection drug users had slightly delayed viral suppression when treated with grazoprevir/elbasvir combination therapy, but this was mainly due to adherence challenges. The finding suggests that stating that adherence issues can delay suppression in some injection drug users. Since the current study lacks on the analysis of treatment adherence, further trials investigating on efficacy of the shortened treatment are needed to confirm the role of injection drug use in viral suppression.

Unexpectedly, HIV co-infection was found to be associated with early viral suppression among cirrhotic patients in this study. Townsend et al.^35^ reported that patients with HIV and hepatitis C co-infection demonstrated a rapid viral decline in response to interferon-free DAA therapy, regardless of cirrhosis status. Conversely, Dumea and Cambrea^36^ found that HIV co-infected cirrhotic patients experienced delayed viral suppression compared to HCV monoinfected individuals, potentially due to immune dysfunction and interactions with HIV antiretroviral therapy (ART). The discrepancy in these findings suggests that multiple factors may influence treatment response. One possible explanation is that HIV-induced immune activation may paradoxically enhance HCV clearance by stimulating inflammatory cytokine release and CD8 + T-cell responses, as suggested by Chew and Bhattacharya^37^. Additionally, HIV/HCV co-infected patients often receive more intensive clinical monitoring and adherence support, which may contribute to improved treatment outcomes. Bruno et al.^38^ found that adherence was a key determinant of viral suppression among HIV/HCV co-infected patients receiving DAAs, which may explain the observed early suppression rates in this subgroup. However, it is also important to note that some studies indicate HIV-driven chronic immune activation can impair HCV clearance, leading to delayed viral suppression in some cohorts^39^. These findings highlight the complexity of HIV/HCV co-infection and its impact on treatment response. Further investigation is needed to determine the clinical significance of these associations and to elucidate the underlying mechanisms driving these observations.

Additionally, our findings indicate that early viral suppression (within 4 or 8 weeks) does not significantly correlate with achieving SVR12, as similar SVR12 rates were observed regardless of suppression timing (*p* > 0.05). This suggests that while early suppression is common, it should not be assumed to predict treatment success. While the results highlight the potential feasibility of a shorter treatment course, they do not confirm its efficacy that reducing treatment duration would yield comparable SVR12 rates to standard regimens. Further clinical trials assessing SVR12 outcomes for a shortened RDV/SOF regimen is necessary before any changes to treatment guidelines can be recommended.

To the best of our knowledge, this is the first study analysing the time to viral load suppression, especially with RDV/SOF treatment for chronic HCV infection; hence, very few comparisons with the current findings could be made. However, there is much evidence on the efficacy and safety of shorter treatment courses for different combination therapies. For example, many studies evaluating shorter treatments of sofosbuvir plus ledipasvir have been performed, and the eight-week course was found to be highly efficacious and well tolerated in chronic hepatitis C patients^40–42^, even in adolescents^43^. Additionally, sofosbuvir-based treatment for eight weeks effectively suppressed HCV infection in 95% of patients with no cirrhosis, with only a 1–2% occurrence of virological failure^44^. Furthermore, the eight-week course of daclatasvir and half-dose sofosbuvir was proven to be efficacious for acute hepatitis C in patients with advanced renal failure^45^. Multiple studies have consistently reported high SVR12 rates of more than 95% for HCV patients receiving eight-week glecaprevir/pibrentasvir regimens across different genotypes and patient populations, including treatment-naïve patients and those with compensated cirrhosis^46–49^. The treatment was well tolerated, with no significant virologic failures observed, and showed similar efficacy to the 12-weeks treatment. Since RDV is still considered one of the newest treatments for chronic hepatitis C infection, no studies have been conducted to assess the efficacy of a shorter RDV/SOF treatment course, hence the need for one.

It is believed that shorter treatment courses for chronic hepatitis C patients offer several benefits. Shorter treatment durations are often associated with better patient adherence^50^. Recent studies have reported that as the duration of treatment increases, patient adherence decreases^51,52^. Apart from improved adherence, shorter treatment courses may be more cost-effective, as they may require fewer resources and reduce overall treatment costs, including medication and monitoring expenses^50^. A study by Morgan et al.^53^ suggest that the 8-week LDV/SOF regimen is more cost-effective than the 12-week course and can achieve superior population-level outcomes across both black and nonblack patient groups. A shortened SOF plus daclatasvir therapy was also proven to decrease the use of DAAs in hepatitis C patients with mild liver disease while still achieving high cure rates and therefore significantly reduced costs, especially in countries where pricing is based on a per-pill basis rather than per treatment course, such as Vietnam and the USA^23,25^.

On the other hand, the potential risks of shorter treatment course also need to be considered. A study comparing 12-week and six-week sofosbuvir/velpatasvir treatments reported that the six-week course had resulted in an inferior cure rate and was not as effective as the standard 12-week course^54^. Another study evaluating the efficacy of an eight-week course of treatment with grazoprevir/elbasvir in HCV-infected MSM patients demonstrated that while the regimen achieved a high SVR12 rate of 96%, resistance-associated substitutions (RAS) were detected in one patient^55^. These findings underscore the potential risks that can emerge, hence the need for rigorous evaluation of shorter treatment approaches. Therefore, while the current findings support the possibility of shorter RDV/SOF treatment, further evidence evaluating the benefits and risks are essential for better overall outcomes, before changing clinical guidelines. Prospective clinical trials would be needed to rigorously evaluate whether shorter treatment durations of RDV/SOF combination can maintain high cure rates without increasing relapse risk, considering factors such as patient adherence, potential resistance development, and post-treatment relapse rates.

## Limitations

Our study is subject to several limitations. Data were collected from public academic and non-academic centers in Malaysia and Thailand, which introduces potential variability due to differences in clinical documentation practices and regional healthcare delivery. While clinical expert supervision aims to validate data consistency, inherent variations in data capture and reporting may impact the study’s generalizability. Patient-reported data may also present additional constraints, due to recall bias, self-reporting errors, incomplete or subjective reporting. For example, participants may experience hesitancy in disclosing sensitive information related to stigmatized topics, including drug and alcohol use, comorbidities such as HIV, and potential lifestyle factors. This potential for underreporting or selective disclosure could introduce bias in key variables and clinical outcomes. Another limitation of our study is the absence of a sensitivity analysis, which could have further validated the robustness of our findings under varying analytical conditions. Besides that, our study lacks on some data regarding important factors like treatment adherence and HIV viral load that may influence viral suppression rates, which limits us from directly assessing their impact. The absence of such data remains a limitation, as these factors could contribute to variations in suppression outcomes. Future studies with more comprehensive datasets, including detailed adherence measures and viral load monitoring, are needed to better understand their role in viral suppression.

## Conclusion

Since the STORM-C-1 study, numerous direct-acting antiviral (DAA) combinations have emerged for HCV treatment. Our analysis revealed that viral load suppression occurs as early as four weeks in most patients, with over 90% suppression by week eight, demonstrating the potential of the RDV/SOF regimen for early viral response. However, this does not necessarily guarantee a sustained virologic response (SVR). While the findings suggest potential for shorter treatment durations, they should not be interpreted as a definitive recommendation for shortened treatment courses. The current study serves as a preliminary exploration of viral suppression patterns rather than a conclusive treatment protocol. Therefore, future research should include large-scale multicentre trials to evaluate the safety and efficacy of shortened treatment regimens before clinical implementation. This includes comprehensive efficacy assessments across diverse patient populations, longitudinal studies to confirm long-term outcomes and SVR, and detailed analyses of factors influencing viral load suppression and treatment success.

## Data Availability

The dataset used and analysed during this study are available upon reasonable request from the corresponding author.
